# Supplementary material for: Parental Preferences for Expanded Newborn Screening: What Are the Limits?
Source: Children (Basel). 2023 Aug 9;10(8):1362. doi: 10.3390/children10081362 (PMC10453746; doi:10.3390/children10081362)
Supplement: Supplementary file 1 [file children-10-01362-s001.zip › children-2414720-supplementary.pdf]

Supplementary Materials 1- Study Survey

Welcome to our study and thank you for taking the time to participate. We are conducting a study on newborn screening and the types of conditions we should include in the newborn screening program.

New technology is being considered for use in newborn screening. This new technology can help improve the accuracy of newborn screening and increase the number of health conditions detected.

We would like your input about the types of health conditions you think we should include in the newborn screening program. Your input will help inform Newborn Screening Ontario and the rest of Canada.

This survey will consist of the following:

-General Questions about you and your family (2-3 minutes)

-Information about newborn screening and the new technology available (5-6 minutes)

-Descriptions of different types of health conditions with questions about whether you would want newborn screening for these types of health conditions (15-20 minutes)

-Questions about your opinions on healthcare (2-3 minutes)

Your rights as a research participant:

Your participation in this research study is entirely voluntary. You are free to decline or withdraw from participation. Any information you provide will be managed in a secure and private manner. Your answers will remain unidentified.

This study has been approved by the Children's Hospital of Eastern Ontario.

Section 1: About You

1. What age group best describes you?

☐Less than 20 years

☐20-29

☐30-39

☐40-49

☐50 or older

2. What gender best describes you?

☐Male

☐Female

☐Gender diverse

☐You don't have an option that applies to me

3. **Are you:**

☐Married

☐Living common-law

☐Widowed

☐Separated

☐Divorced

☐Single-never married

4. What is the highest degree, certificate or diploma you have obtained?

☐No post-secondary degree, certificate or diploma

☐Trade certificate or diploma from a vocational school or apprenticeship training

☐Non-university certificate or diploma from a community college

☐University certificate below bachelor's level

☐Bachelor's degree

☐University degree or certificate above bachelor's degree

5. Which of the following best describes where you live?

☐Rural area

☐Small city/town (less than 100,000 people)

☐Medium-sized city (100,000-499,999 people)

☐Large city (500,000 or more people)

6. How many children do you have?

☐One

☐Two

☐Three

☐Four

☐Five or more

7. Have any of your children received a positive screening result or a confirmed diagnosis through newborn screening to date?

☐Yes

☐No

☐I don't recall

☐If yes, what condition(s) ___________________________

8. Has anyone in your immediate family received genetic testing for an inherited condition?

☐Yes

☐No

☐If yes, what condition(s) ___________________________

Section 2: Information about Newborn Screening

We recognize that it may have been some time since you have learned about newborn screening. The following questions are about your understanding of newborn screening. Don't worry about getting these questions correct!

	True	False
The main purpose of newborn screening to identify infants who may have a disease and to provide treatment right away to prevent serious health problems.		
An infant can receive a positive newborn screening test result even if he/she does not have the disease.		
Uncertain results in newborn screening mean that doctors may not know if your infant has the condition for months or years		
New newborn screening technology may detect conditions that cannot be treated		
A parent can decline newborn screening for their infant?		

What is newborn screening?

In the first few days after birth, a few drops of blood are taken from the baby's heel and collected on a card. This blood sample is sent to a laboratory which studies the samples for signs of several rare and serious health conditions. This testing is important because most babies seem healthy when they are born, but some of them may have one of these health conditions.

The goal of newborn screening is to identify babies who have one of these health conditions so that they can receive treatment before serious health problems occur. Starting treatment before the onset of symptoms can improve the child's health outcomes.

The health problems associated with the conditions that we test for in newborn screening include one or more of the following:

- Sudden death, or shortened life expectancy
- Health problems related to the baby's growth, digestion, breathing and muscle strength
- Pain and discomfort
- Intellectual disability

As a public health program, newborn screening tries to test ALL babies at birth to identify babies who need treatment. This testing is not required but considered routine unless a parent declines testing.

What types of results are generated by newborn screening?

<u>Screen negative result</u>

- The baby was not found to be at an increased chance of having the conditions screened for on newborn screening. Parents are not contacted if there is a negative result.

<u>Screen positive result</u>

- The baby has a **higher chance** of having a disease
- The baby will **need further testing** to see if they have the disease

- After further testing, the baby can receive:
    - **A false-positive result:** The baby does not have the disease

      A true-positive result: The baby is confirmed to have the disease. This is also known as a diagnosis.

- **An uncertain result:** We do not know if the baby has the disease. We may not learn if the baby has the condition for many months or sometimes years. These types of results can lead to multiple doctor’s visits for the baby, which in the end, the child may or may not have needed. These doctor’s visits may allow for early detection of health problems related to the disease.

Technology used in newborn screening

The technology we currently use in newborn screening looks in baby’s blood for markers of various health conditions

There is a new technology that can be used in newborn screening. This technology looks for DNA, or the instruction manuals of the body, in the baby's blood. This new technology is still being tested, but it can detect a broader range of genetic diseases. This includes diseases which may not have treatment or develop symptoms until later on in life

Section 3: Your Preferences about Different Conditions

To help NBS programs and governments make decisions about using new NBS technologies, we would like to understand how parents weigh their risks and benefits.

In this section, you will be presented with different health conditions that the new technology could pick up. Not all of these conditions are currently treatable and some of them develop at an older age.

Please read the description of each condition and think about:

1) *Whether you would want this type of condition to be identified by newborn screening, using new technology.*
2) *Whether you would like to have a choice about receiving newborn screening results for this type of condition.*
   Currently in Ontario, a healthcare provider informs parents about newborn screening prior to the test. Unless the parent opts out of receiving the test, it is presumed that they want to receive the test for their baby. As new types of conditions are added to newborn screening, the approach to parent choice (i.e. informed consent) might change.

Scenario #1

Imagine there was a rare condition where a child has serious breathing problems, weakness in their arms and legs, hearing loss, and vision problems. The breathing problems are the most serious symptoms, and may cause a child to need machine assistance for breathing, or even cause death. Although this condition usually causes symptoms in the first year of life, some children may not show symptoms until later in childhood.

There is treatment available for this condition after the child is diagnosed with the condition. Treatment for this condition can be lifesaving by preventing serious breathing problems and can also improve symptoms in other parts of the body.

Please select the answer which matches your opinion.

1) Newborn screening for this condition would be beneficial for the child.

5<br>Strongly Agree	4<br>Agree	3<br>Neither Agree or Disagree	2<br>Disagree	1<br>Strongly Disagree

2) Including a condition in newborn screening before the child has symptoms would interfere with parent-child bonding.

5<br>Strongly Agree	4<br>Agree	3<br>Neither Agree or Disagree	2<br>Disagree	1<br>Strongly Disagree

3) Receiving a true-positive result from newborn screening for this condition would lead to better support for the child and their parents.

5<br>Strongly Agree	4<br>Agree	3<br>Neither Agree or Disagree	2<br>Disagree	1<br>Strongly Disagree

4) Receiving a true-positive result from newborn screening for this condition would cause unnecessary parental anxiety.

5<br>Strongly Agree	4<br>Agree	3<br>Neither Agree or Disagree	2<br>Disagree	1<br>Strongly Disagree

5) Receiving an uncertain result from newborn screening for this condition would lead to better support for the child and their parents.

5<br>Strongly Agree	4<br>Agree	3<br>Neither Agree or Disagree	2<br>Disagree	1<br>Strongly Disagree

6) Receiving an uncertain result from newborn screening for this condition would cause unnecessary parental anxiety.

5<br>Strongly Agree	4<br>Agree	3<br>Neither Agree or Disagree	2<br>Disagree	1<br>Strongly Disagree

7) Newborn screening for this condition could help parents with decision-making regarding future pregnancies.

5<br>Strongly Agree	4<br>Agree	3<br>Neither Agree or Disagree	2<br>Disagree	1<br>Strongly Disagree

8) Newborn screening for this condition would lead to labelling or stigma for the child.

5<br>Strongly Agree	4<br>Agree	3<br>Neither Agree or Disagree	2<br>Disagree	1<br>Strongly Disagree

9) I would want my baby screened for a health condition like this through newborn screening

☐Yes ☐No

As you may recall, newborn screening in Ontario is routine, meaning every baby receives newborn screening unless a parent declines. For this condition, parents should be…

… required to have their baby screened. This means that parents should not be able to decline testing for this condition.

☐Yes ☐No

…strongly encouraged to have their baby screened. This means that parents will be advised by their healthcare providers to have their baby screened for this condition. Parents can still decline to have their baby to be screened.

☐Yes ☐No

…able to choose whether they want their baby screened. This means that parents can decline to have their baby screened for this condition.

☐Yes ☐No

If there was an informed consent process for this condition, when should it happen?

☐ Early in pregnancy (first or second trimester) ☐ Late in pregnancy (third trimester)

☐ When the baby is born ☐ When the sample is about to be taken (i.e. 48-72 hours after birth)

Scenario # 2

Imagine there was a rare condition where the child has normal development early on, but at around age 2, they start having problems with walking and problems with talking. Children with this condition have seizures, learning challenges, and problems with their muscles and bones. Most children with this condition live into adulthood.

There is currently no treatment for this condition. Knowing about this condition at birth can help with early access to supportive services that may help the child learn to move and talk better. These services may also help parents with strategies for how to best care for their child to help them reach their full potential.

However, there is a concern about giving a child a diagnosis before they show symptoms. This may have impacts on family bonding with the child or cause undue distress for the family. Some families may feel like they are waiting for symptoms to appear by giving their child a medical label at birth.

Please select the answer which matches your opinion.

1) Newborn screening for this condition would be beneficial for the child.

5<br>Strongly Agree	4<br>Agree	3<br>Neither Agree or Disagree	2<br>Disagree	1<br>Strongly Disagree

2) Including a condition in newborn screening before the child has symptoms would interfere with parent-child bonding.

5<br>Strongly Agree	4<br>Agree	3<br>Neither Agree or Disagree	2<br>Disagree	1<br>Strongly Disagree

3) Receiving a true-positive result from newborn screening for this condition would lead to better support for the child and their parents.

5<br>Strongly Agree	4<br>Agree	3<br>Neither Agree or Disagree	2<br>Disagree	1<br>Strongly Disagree

4) Receiving a true-positive from newborn screening for this condition would cause unnecessary parental anxiety.

5<br>Strongly Agree	4<br>Agree	3<br>Neither Agree or Disagree	2<br>Disagree	1<br>Strongly Disagree

5) Receiving an uncertain result from newborn screening for this condition would lead to better support for the child and their parents.

5<br>Strongly Agree	4<br>Agree	3<br>Neither Agree or Disagree	2<br>Disagree	1<br>Strongly Disagree

6) Receiving an uncertain result from newborn screening for this condition would cause unnecessary parental anxiety.

5<br>Strongly Agree	4<br>Agree	3<br>Neither Agree or Disagree	2<br>Disagree	1<br>Strongly Disagree

7) Newborn screening for this condition could help parents with decision-making regarding future pregnancies

5<br>Strongly Agree	4<br>Agree	3<br>Neither Agree or Disagree	2<br>Disagree	1<br>Strongly Disagree

8) Newborn screening for this condition would lead to labelling or stigma for the child.

5<br>Strongly Agree	4<br>Agree	3<br>Neither Agree or Disagree	2<br>Disagree	1<br>Strongly Disagree

9) I would want my baby screened for a health condition like this through newborn screening
☐ Yes ☐ No

As you may recall, newborn screening in Ontario is routine, unless a parent declines. For this condition, parents should be…

… required to have their baby screened. This means that parents should not be able to decline testing for this condition.

☐ Yes ☐ No

…strongly encouraged to have their baby screened. This means that parents will be advised by their healthcare providers to have their baby screened for this condition. Parents can still decline to have their baby to be screened.

☐ Yes ☐ No

…able to choose whether they want their baby screened. This means that parents can decline to have their baby screened for this condition.

☐ Yes ☐ No

If there was an informed consent process for this condition, when should it happen?

☐ Early in pregnancy (first or second trimester) ☐ Late in pregnancy (third trimester)

☐ When the baby is born ☐ When the sample is about to be taken (i.e. 48-72 hours after birth)

Scenario 3

Imagine there was a rare condition where the child's muscles gradually get weaker over time. The child would start having problems walking around age 4. Over time, the child would need to use a wheelchair to help move around. Heart problems could develop that are usually the cause of death at ~age 30.

There is currently no treatment or cure for this condition There are medications to treat symptoms as they arise. Researchers are trying to develop new medications to treat or cure this condition. Having a diagnosis early may allow children to participate in research for this condition and potentially benefit from a new treatment. However, it is unknown how long it will take to develop this treatment/cure.

Some families have concerns about giving a child a diagnosis before they show symptoms. This may impact family bonding with the child. It can also cause undue distress for the family to learn that their child will develop symptoms which get worse over time, when there is not a cure available.

Please select the answer which matches your opinion.

1) Newborn screening for this condition would be beneficial for the child.

5<br>Strongly Agree	4<br>Agree	3<br>Neither Agree or Disagree	2<br>Disagree	1<br>Strongly Disagree

2) Including a condition in newborn screening before the child has symptoms would interfere with parent-child bonding.

5<br>Strongly Agree	4<br>Agree	3<br>Neither Agree or Disagree	2<br>Disagree	1<br>Strongly Disagree

3) Receiving a true-positive result from newborn screening for this condition would lead to better support for the child and their parents.

5<br>Strongly Agree	4<br>Agree	3<br>Neither Agree or Disagree	2<br>Disagree	1<br>Strongly Disagree

4) Receiving a true-positive result from newborn screening for this condition would cause unnecessary parental anxiety.

5<br>Strongly Agree	4<br>Agree	3<br>Neither Agree or Disagree	2<br>Disagree	1<br>Strongly Disagree

5) Receiving an uncertain result from newborn screening for this condition would lead to better support for the child and their parents.

5<br>Strongly Agree	4<br>Agree	3<br>Neither Agree or Disagree	2<br>Disagree	1<br>Strongly Disagree

6) Receiving an uncertain result from newborn screening for this condition would cause unnecessary parental anxiety.

5<br>Strongly Agree	4<br>Agree	3<br>Neither Agree or Disagree	2<br>Disagree	1<br>Strongly Disagree

7) Newborn screening for this condition could help parents with decision-making regarding future pregnancies

5<br>Strongly Agree	4<br>Agree	3<br>Neither Agree or Disagree	2<br>Disagree	1<br>Strongly Disagree

8) Newborn screening for this condition would lead to labelling or stigma for the child.

5<br>Strongly Agree	4<br>Agree	3<br>Neither Agree or Disagree	2<br>Disagree	1<br>Strongly Disagree

7) I would want my baby be screened for a health condition like this through newborn screening

☐ Yes ☐ No

As you may recall, newborn screening in Ontario is routine, unless a parent declines. For this condition, parents should be…

… required to have their baby screened. This means that parents should not be able to decline testing for this condition.

☐ Yes ☐ No

…strongly encouraged to have their baby screened. This means that parents will be advised by their healthcare providers to have their baby screened for this condition. Parents can still decline to have their baby to be screened.

☐ Yes ☐ No

…able to choose whether they want their baby screened. This means that parents can decline to have their baby screened for this condition.

☐ Yes ☐ No

If there was an informed consent process for this condition, when should it happen??

☐ Early in pregnancy (first or second trimester) ☐ Late in pregnancy (third trimester)

☐ When the baby is born ☐ When the sample is about to be taken (i.e. 48-72 hours after birth)

Scenario 4

Imagine there was screening for a heart condition that causes shortness of breath, abnormal heartbeat, chest pain and heart failure. A small number of individuals with this condition can

have a sudden heart attack while other individuals with this condition may never develop symptoms in their lifetime. This condition usually starts in adolescence or adulthood but can also start in childhood.

There is no cure for this condition. Knowing about this heart problem through newborn screen may lead to regular appointments to monitor the heart. Monitoring may or may not reduce the risk of more serious problems heart problems in the future. Knowing about this health risk early could also lead doctors to restrict your child from playing certain sports.

Please select the answer which matches your opinion.

1) Newborn screening for this condition would be beneficial for the child.

5<br>Strongly Agree	4<br>Agree	3<br>Neither Agree or Disagree	2<br>Disagree	1<br>Strongly Disagree

2) Including a condition in newborn screening before the child has symptoms would interfere with parent-child bonding.

5<br>Strongly Agree	4<br>Agree	3<br>Neither Agree or Disagree	2<br>Disagree	1<br>Strongly Disagree

3) Receiving a true-positive result from newborn screening for this condition would lead to better support for the child and their parents.

5<br>Strongly Agree	4<br>Agree	3<br>Neither Agree or Disagree	2<br>Disagree	1<br>Strongly Disagree

4) Receiving a true-positive result from newborn screening for this condition would cause unnecessary parental anxiety.

5<br>Strongly Agree	4<br>Agree	3<br>Neither Agree or Disagree	2<br>Disagree	1<br>Strongly Disagree

5) Receiving an uncertain result from newborn screening for this condition would lead to better support for the child and their parents.

5<br>Strongly Agree	4<br>Agree	3<br>Neither Agree or Disagree	2<br>Disagree	1<br>Strongly Disagree

6) Receiving an uncertain result from newborn screening for this condition would cause unnecessary parental anxiety.

5<br>Strongly Agree	4<br>Agree	3<br>Neither Agree or Disagree	2<br>Disagree	1<br>Strongly Disagree

7) Newborn screening for this condition could help parents with decision-making regarding future pregnancies

5<br>Strongly Agree	4<br>Agree	3<br>Neither Agree or Disagree	2<br>Disagree	1<br>Strongly Disagree

8) Newborn screening for this condition creates would lead to labelling or stigma for the child.

5<br>Strongly Agree	4<br>Agree	3<br>Neither Agree or Disagree	2<br>Disagree	1<br>Strongly Disagree

9) I would want my baby be screened for a health condition like this through newborn screening

☐ Yes ☐ No

As you may recall, newborn screening in Ontario is routine, unless a parent declines. For this condition, parents should be…

… required to have their baby screened. This means that parents should not be able to decline testing for this condition.

☐ Yes ☐ No

…strongly encouraged to have their baby screened. This means that parents will be advised by their healthcare providers to have their baby screened for this condition. Parents can still decline to have their baby to be screened.

☐ Yes ☐ No

…able to choose whether they want their baby screened. This means that parents can decline to have their baby screened for this condition.

☐ Yes ☐ No

If there was an informed consent process for this condition, when should it happen?

☐ Early in pregnancy (first or second trimester) ☐ Late in pregnancy (third trimester)

☐ When the baby is born ☐ When the sample is about to be taken (i.e. 48-72 hours after birth)

Section 4: Your Attitudes toward Healthcare

Now we'd like to ask you some general questions about health care in Canada. **To what extent do you agree or disagree with the following statements:**

1. **The government ensures a high-quality health care system.** *(Please check only one)*

☐	☐	☐	☐	☐
Strongly agree	Agree	Neutral	Disagree	Strongly disagree

2. **If the government has funded a medical test or procedure, it is probably a worthwhile test to have.** *(Please check only one)*

☐	☐	☐	☐	☐
Strongly agree	Agree	Neutral	Disagree	Strongly disagree

3. **I think there are medical tests and/or treatments that the government should fund, but they haven't.** *(Please check only one)*

☐	☐	☐	☐	☐
Strongly agree	Agree	Neutral	Disagree	Strongly disagree

4. **The government wouldn't fund a medical test or procedure if they were not sure of its benefits.** *(Please check only one)*

☐	☐	☐	☐	☐
Strongly agree	Agree	Neutral	Disagree	Strongly disagree

Supplementary Materials 2 -Additional Figures

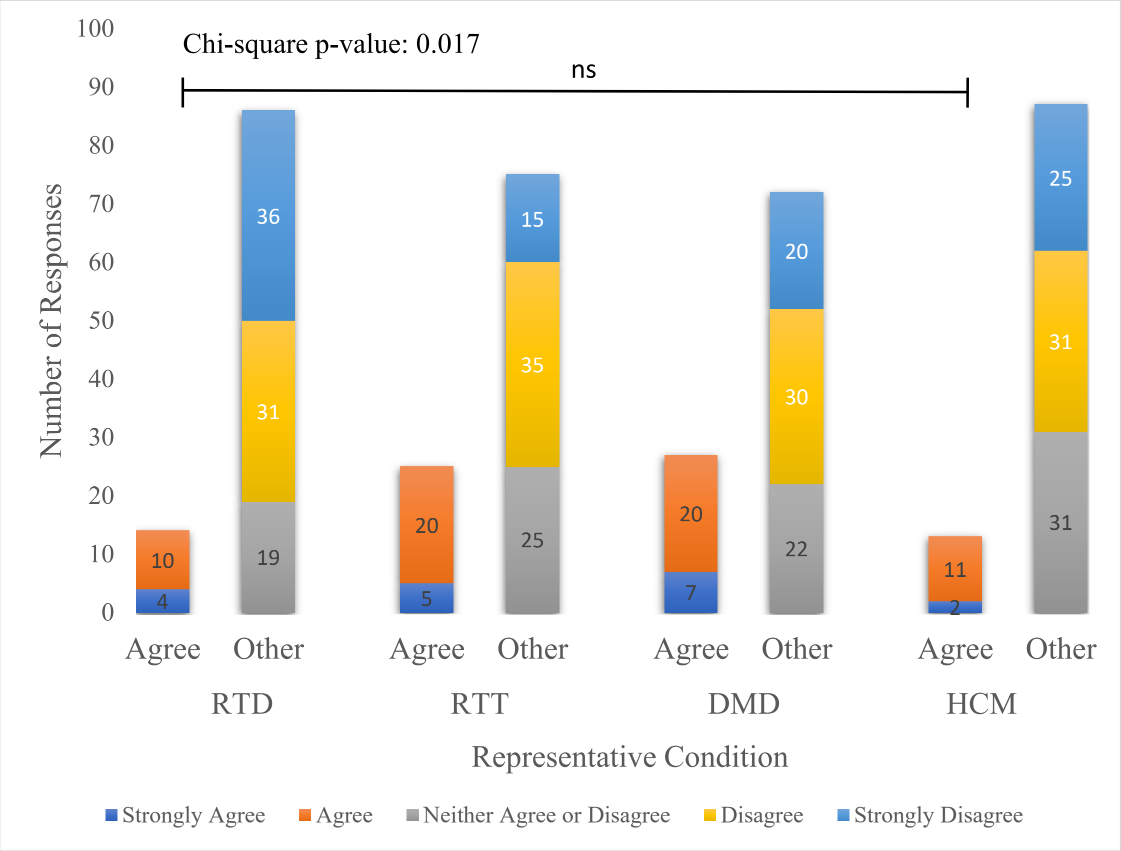

Supplementary Figure S1. Participant responses to the attitude statement "Including a condition in newborn screening before the child has symptoms would interfere with parent-child bonding." by condition category. 'Agree' includes participants who selected 'Strongly Agree' or 'Agree'. RTD = riboflavin transporter deficiency; RTT = Rett syndrome; DMD = Duchenne muscular dystrophy; HCM= Hypertrophic cardiomyopathy. Asterisks indicate statistically significant pairwise comparisons between representative conditions.

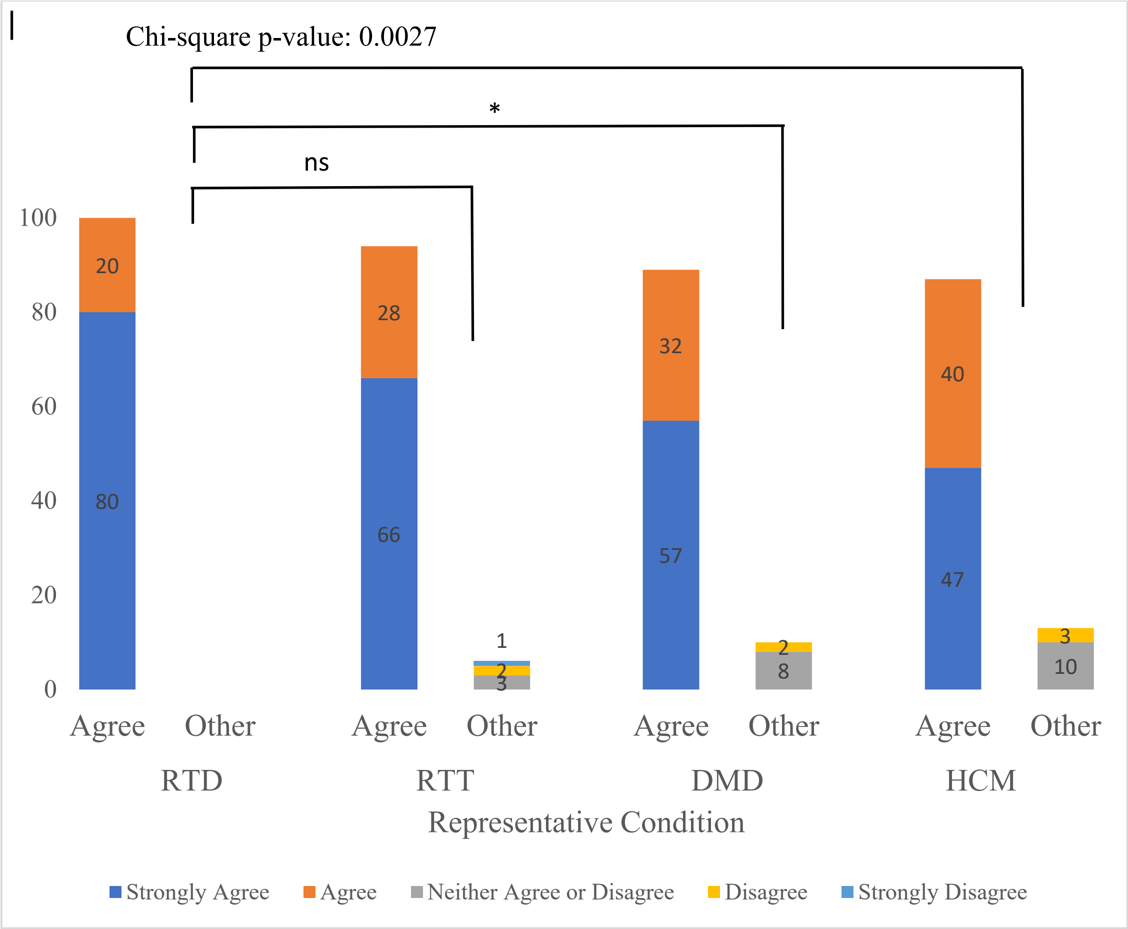

Supplementary Figure S2. Participant responses to the attitude statement "Receiving a true-positive result from newborn screening for this condition would lead to better support for the child and their parents." by condition category. 'Agree' includes participants who selected 'Strongly Agree' or 'Agree'. Asterisks indicate statistically significant pairwise comparisons between representative conditions.

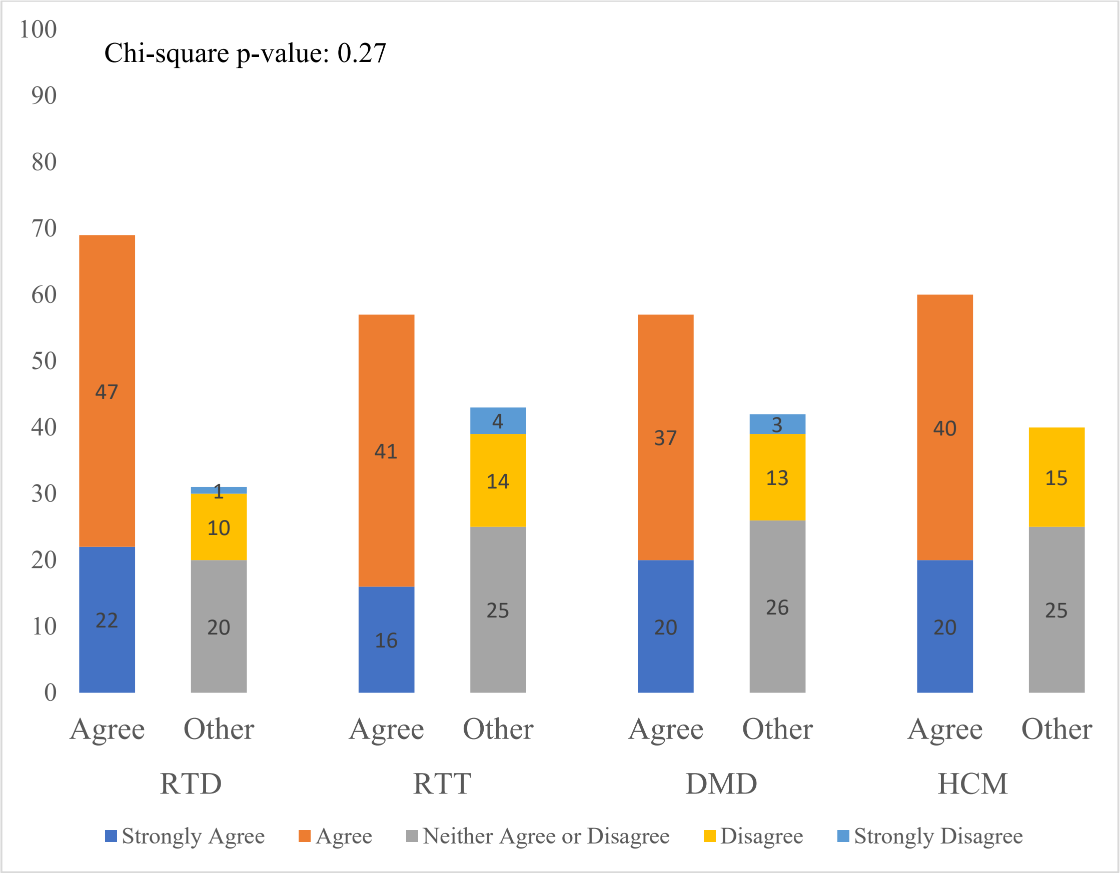

Supplementary Figure S3. Participant responses to the attitude statement "Receiving an uncertain result from newborn screening for this condition would lead to better support for the child and their parents." by condition category. 'Agree' includes participants who selected 'Strongly Agree' or 'Agree'.

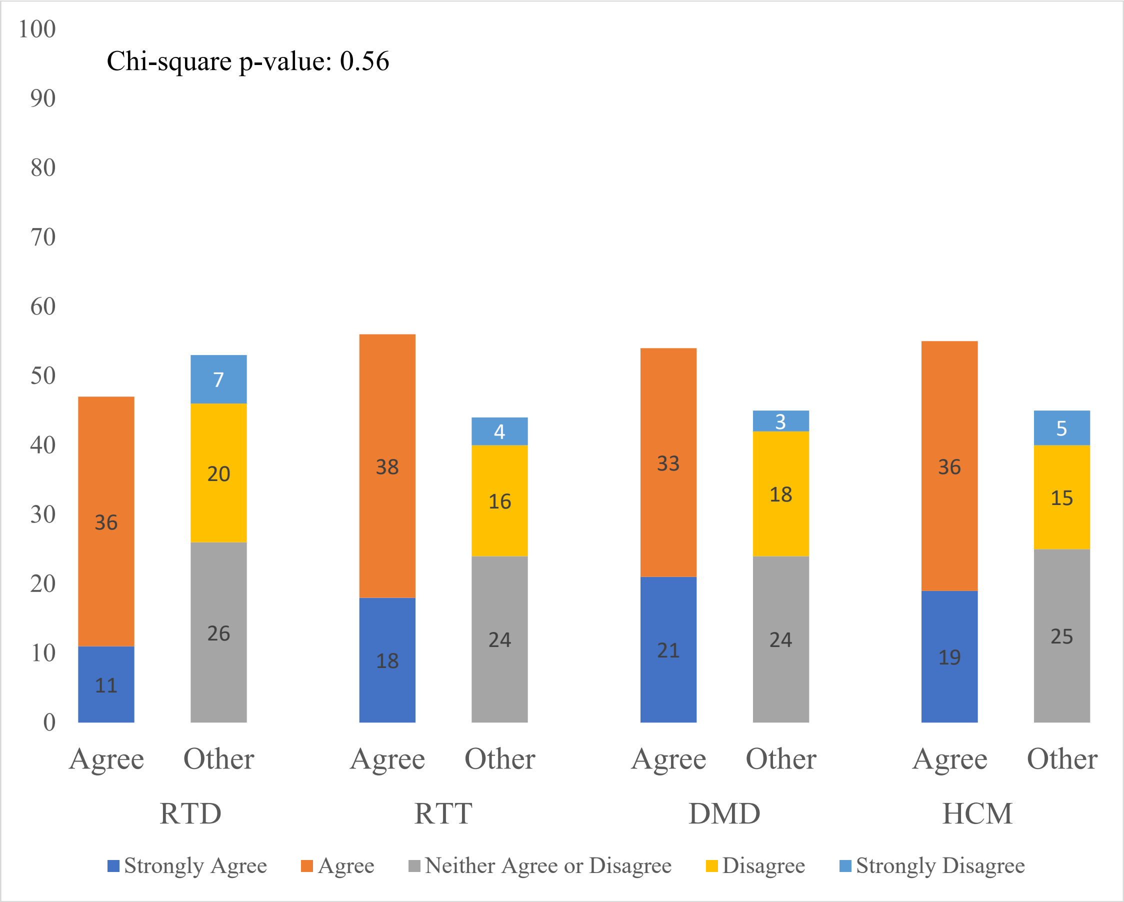

Supplementary Figure S4. Participant responses to the attitude statement "Receiving an uncertain result from newborn screening for this condition would cause unnecessary parental anxiety." by condition category. ‘Agree’ includes participants who selected ‘Strongly Agree’ or ‘Agree’.

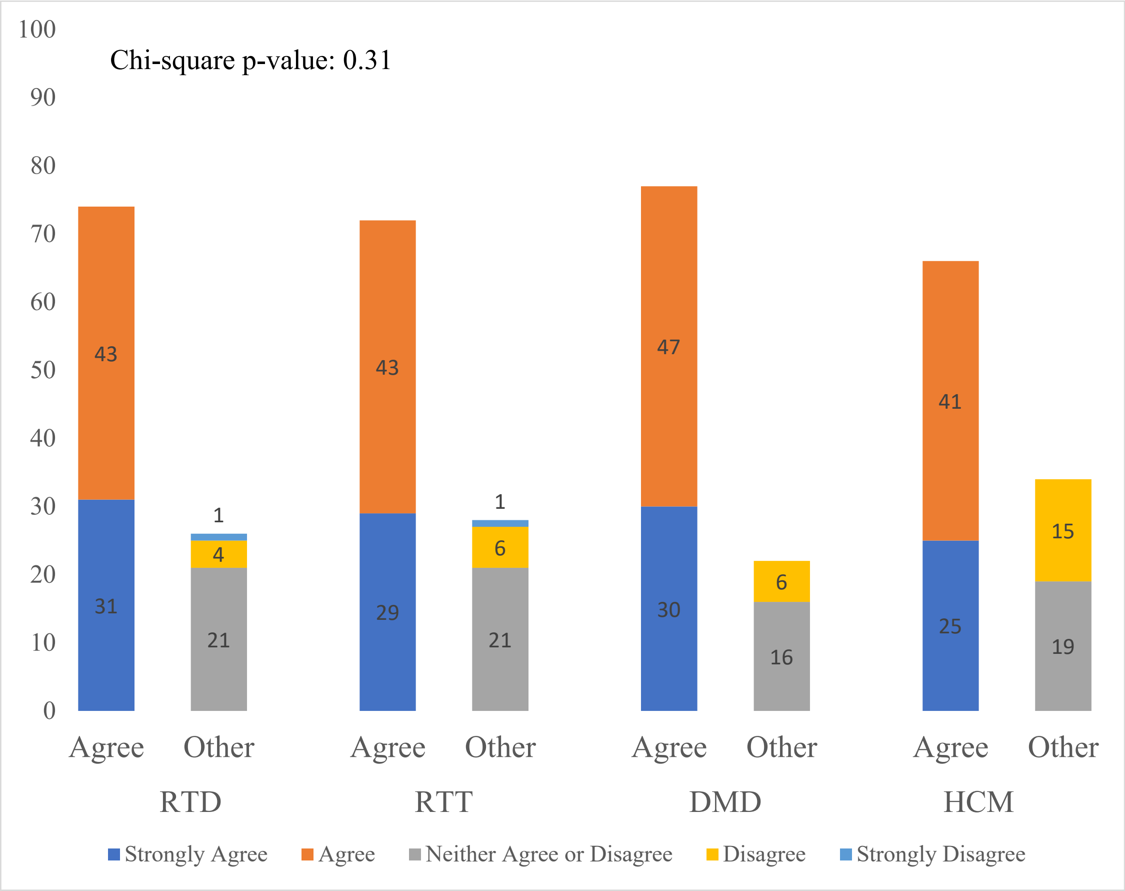

Supplementary Figure S5. Participant responses to the attitude statement "Newborn screening for this condition could help parents with decision-making regarding future pregnancies." by condition category. 'Agree' includes participants who selected 'Strongly Agree' or 'Agree'.

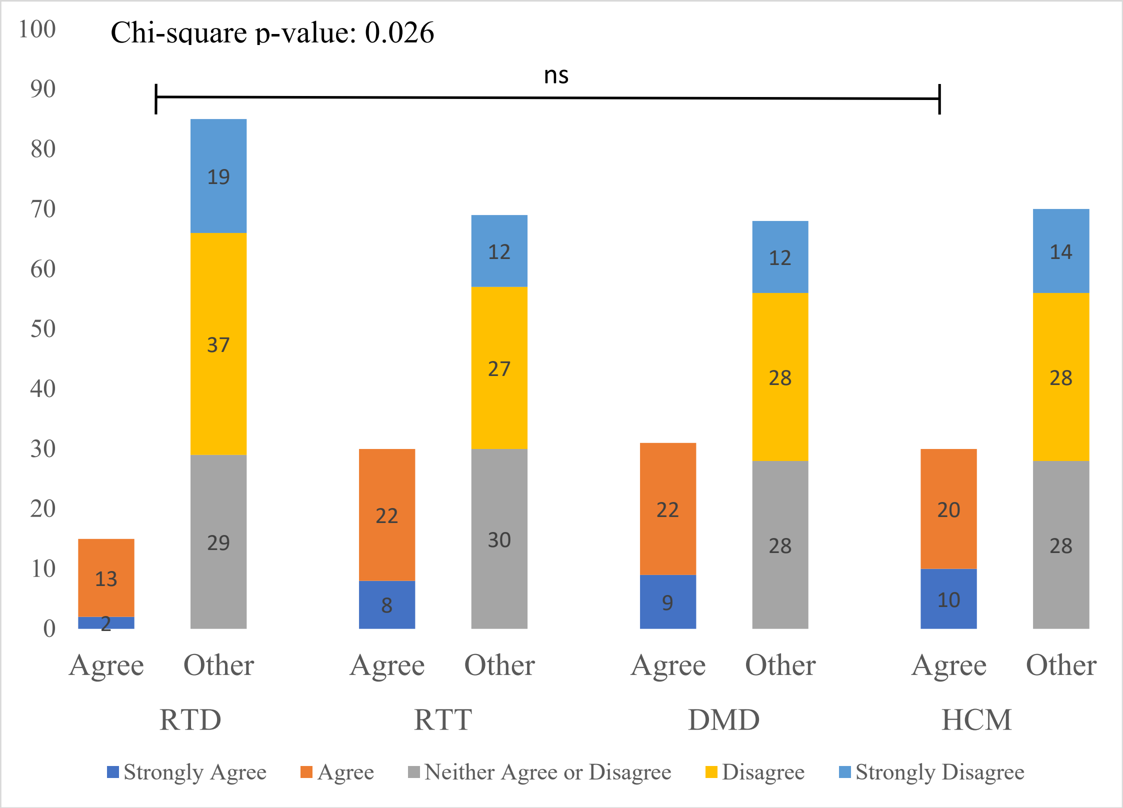

Supplementary Figure S6. Participant responses to the attitude statement "Newborn screening for this condition would lead to labelling or stigma for the child." by condition category. ‘Agree’ includes participants who selected ‘Strongly Agree’ or ‘Agree’.
